# Functional Analysis of the Dioxin Response Elements (DREs) of the Murine *CYP1A1* Gene Promoter: Beyond the Core DRE Sequence

**DOI:** 10.3390/ijms15046475

**Published:** 2014-04-16

**Authors:** Shuaizhang Li, Xinhui Pei, Wen Zhang, Heidi Qunhui Xie, Bin Zhao

**Affiliations:** 1 Research Center for Eco-Environmental Sciences, Chinese Academy of Sciences, Beijing 100085, China; E-Mails: shuaizhangli@126.com (S.L.); peixinhui@gmail.com (X.P.); qhxie@rcees.ac.cn (H.Q.X.); 2 Foreign Economic Cooperation Office, Ministry of Environmental Protection, Beijing 100035, China; E-Mail: zhang.wen@mepfeco.org.cn

**Keywords:** aryl hydrocarbon receptor (AhR), dioxin responsive element (DRE), 2,3,7,8-tetrachlorodibenzo-*p*-dioxin (TCDD), transcriptional regulation

## Abstract

The aryl hydrocarbon receptor (AhR) is a ligand-dependent transcription factor that mediates the biological and toxicological effects of halogenated aromatic hydrocarbons, such as 2,3,7,8-tetrachlorodibenzo-*p*-dioxin (TCDD). When activated by dioxin, the cytosolic AhR protein complex translocates into the nucleus and dimerizes with the ARNT (Ah receptor nuclear translocator) protein. The heteromeric ligand:AhR/Arnt complex then recognizes and binds to its specific DNA recognition site, the dioxin response element (DRE). DREs are located upstream of cytochrome P4501A1 (*CYP1A1*) and other AhR-responsive genes, and binding of the AhR complex stimulates their transcription. Although *CYP1A1* expression has been used as the model system to define the biochemical and molecular mechanism of AhR action, there is still limited knowledge about the roles of each of the seven DREs located in the CYP1A1 promoter. These seven DREs are conserved in mouse, human and rat. Deletion analysis showed that a single DRE at −488 was enough to activate the transcription. Truncation analysis demonstrated that the DRE at site −981 has the highest transcriptional efficiency in response to TCDD. This result was verified by mutation analysis, suggesting that the conserved DRE at site −981 could represent a significant and universal AhR regulatory element for *CYP1A1*. The reversed substituted intolerant core sequence (5′-GCGTG-3′ or 5′-CACGC-3′) of seven DREs reduced the transcriptional efficiency, which illustrated that the adjacent sequences of DRE played a vital role in activating transcription. The core DRE sequence (5′-TNGCGTG-3′) tends to show a higher transcriptional level than that of the core DRE sequence (5′-CACGCNA-3′) triggered by TCDD. Furthermore, in the core DRE (5′-TNGCGTG-3′) sequence, when “N” is thymine or cytosine (T or C), the transcription efficiency was stronger compared with that of the other nucleotides. The effects of DRE orientation, DRE adjacent sequences and the nucleotide “N” in the core DRE (5′-TNGCGTG-3′) sequence on the AhR-regulated *CYP1A1* transcription in response to TCDD were studied systematically, and our study laid a good foundation for further investigation into the AhR-dependent transcriptional regulation triggered by dioxin and dioxin-like compounds.

## 1. Introduction

Dioxins are typical persistent organic pollutants and one of the 12 most harmful organic pollutants included in the Stockholm convention list. Exposure to dioxin and dioxin-like compounds can produce a variety of biological and toxicological effects, including carcinogenicity, immune toxicity, liver toxicity, dermal toxicity, endocrine disturbance and fertility disorders [1–6]. Dioxin toxic effects are mainly mediated by the activation of the aromatic hydrocarbon receptor (AhR), a basic helix-loop-helix-PAS (bHLH-PAS)-containing transcriptional factor [7]. Numerous studies have shown that some synthetic or natural compounds with different structure are also AhR ligands, indicating a high degree of the structural diversity of AhR ligands [8,9].

In the absence of a ligand, the AhR is mainly localized in the cytoplasm complexed with heat shock protein 90 (hsp90) and several other proteins, including AIP/ARA9/XAP2 ligands [10,11]. Upon ligand binding and activation, the complex undergoes a conformation change, exposing its nuclear localization sequence, which allows it to translocate into the nucleus [12,13]. In the nucleus, the AhR dimerizes with ARNT (aryl hydrocarbon receptor nuclear translocator). Then, the heterodimer recognizes and binds with high affinity to dioxin responsive elements (DREs) in the promoter regions of target genes [14–16]. Since DREs are found in the upstream region of most dioxin-inducible genes, the mere presence of a DRE sequence can be used to judge whether a gene is by AhR [17,18]. The DRE consensus sequence is 5′-TNGCGTG-3′, where the “N” stands for any one of the four nucleotides [19]. Sequences adjacent to the core consensus DRE are also very important, because a cloned DRE alone does not drive reporter gene expression [20,21].

The expression level of *CYP1A1* mRNA was verified to be [22] via the dioxin-responsive receptor-enhancer system [23]. The 2,3,7,8-tetrachlorodibenzo-*p*-dioxin (2,3,7,8-TCDD)-receptor complex interacts directly related to dioxin exposure at molecular and cellular levels [24,25]. The dioxin induction of *CYP1A1* mRNA, encoding a microsomal enzyme, is one element of an adaptive detoxification process that functions by oxygenating aromatic hydrocarbons. In mouse hepatoma cells, 2,3,7,8-tetrachlorodibenzo-*p*-dioxin (TCDD) induces hydroxylase activity by increasing the rate of transcription of *CYP1A1* specifically with DREs [26]. The dioxin-responsive enhancer upstream of the *CYP1A1* contains four DREs, which all contribute to the response of the enhancer to TCDD [27]. There are eight putative DREs in the promoter region of the mouse *CYP1A1*, which are respectively at position −488, −821, −892, −981, −1058, −1203, −1379, −9738 [19,28,29], and we selected seven of them for detailed investigations. In the present study, we characterized these seven DREs dependent on their transcriptional efficiency involving their direction, adjacent sequences and the contribution of the nucleotide “N” in the core DRE (5′-TNGCGTG-3′) using a reporter gene system.

## 2. Results

### 2.1. DREs Conserved in Mouse, Human and Rat

**Figure 1.**
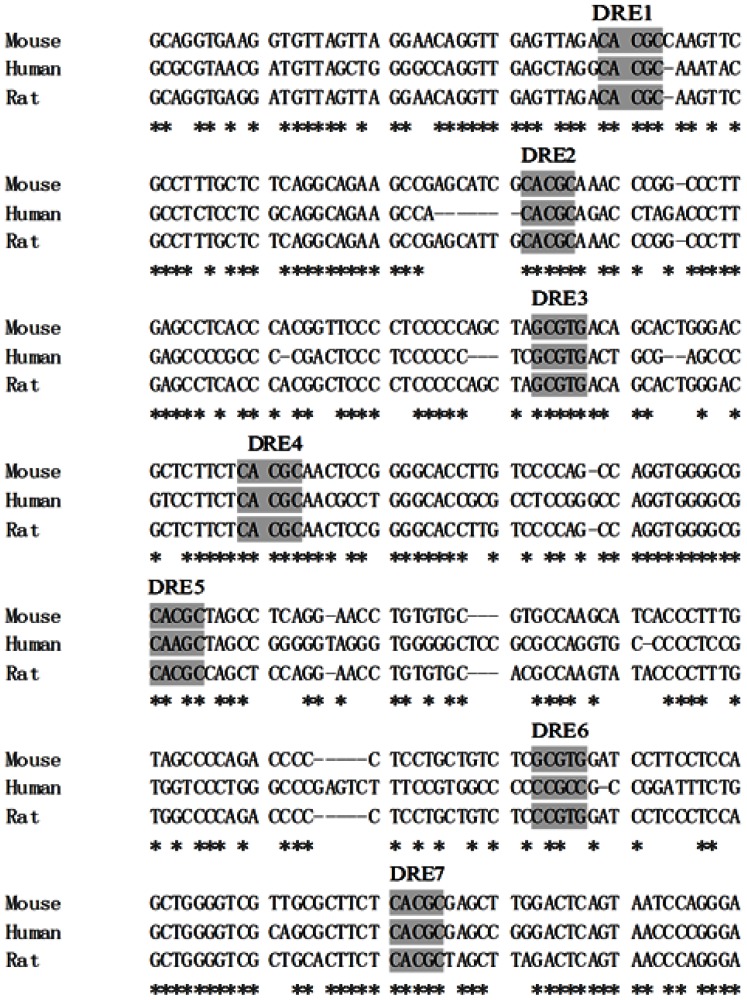
Sequence of the putative dioxin responsive element (DRE) sites on the promoter region of mouse CYP1A1, and the alignments with the corresponding human and rat *CYP1A1* promoter sequences are shown. The asterisk “^*^” indicates positions which have a single, fully conserved residue.

The presence of DREs in the regulatory region upstream of the *CYP1A1* is a key component of the AhR-dependent signaling pathway in response to dioxin. Using software (BioEdit), the 1.4 kb upstream of the *CYP1A1* sequences from mouse, human and rat were aligned. We found that five DREs were conserved among the three species, and two additional DREs (DRE5 and DRE6) were conserved between mouse and rat (Figure 1). In addition, the sequences around DRE1, DRE4, DRE5 and DRE7 were also conserved, implying that they may have the greatest effect on the TCDD induction of *CYP1A1* in human, mouse and rat. It has been reported that two DREs (mouse DRE4 and DRE7) are fully conserved in the position and sequence with the cluster of human DREs, and both of them show AhR binding activity *in vitro* [29]. In addition, the regulatory regions flanking the human and mouse dioxin-responsive receptor-enhancer regions may contribute significantly toward differences in expression patterns, as determined by induction [30].

### 2.2. Single DRE at Position −488 Is Enough to Activate Transcription in Response to Dioxin

**Figure 2.**
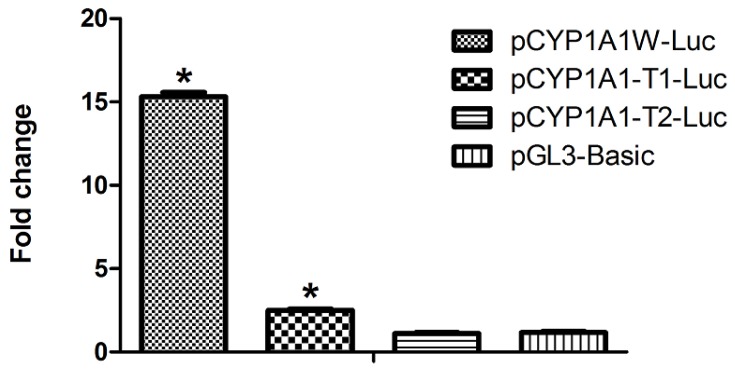
The putative DRE at position −488 is enough to activate AhR-dependent transcription. The mouse CYP1A1 promoter-reporter constructs, pCYP1A1W-Luc, which contains 1.4 kb of the promoter sequence, pCYP1A1-T1-Luc, which contains only one DRE at position −488, and pCYP1A1-T2-Luc, which contains no DREs, were transiently transfected into cultured Hepa wide-type (WT) cells one day before treatment. The transfected cells were incubated with 2,3,7,8-tetrachlorodibenzo-*p*-dioxin (TCDD) (10^−9^ M) or with solvent alone at 0.1% (control). After 24 h, luciferase assays were performed to determine the promoter activity of mouse CYP1A1. ^*^ *p* < 0.05, the difference from solvent-treated cells (control) by one-way ANOVA with Tukey’s test.

In cultures transfected with the ~1.4 kb region upstream of the mouse *CYP1A1* gene, containing seven DRE sites (pCYP1A1W-Luc), we could see that the luciferase activity in TCDD-treated cells was about 15 times higher than that of the control cells treated with solvent alone. The deletion mutant (pCYP1A1-T1-Luc), which contains only DRE at position −488, showed approximately two-fold induction. In the construct without DREs, no induction was found in response to TCDD. The pGL3-basic vector is used as the negative control (Figure 2). The results show that one DRE is sufficient to confer a transcriptional response to TCDD, which lays a good foundation for our following study.

### 2.3. Seven DREs Have Different Transcriptional Efficiency Induced by TCDD

**Figure 3.**
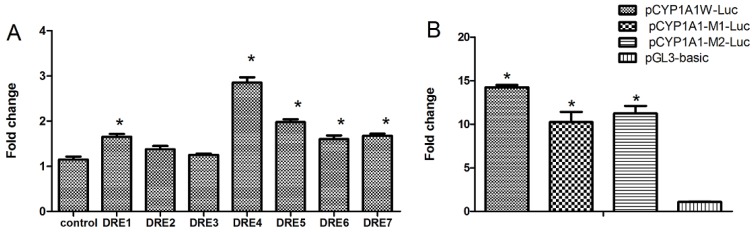
The AhR-dependent transcriptional efficiency of the seven putative DREs are different. The following constructs were transiently transfected into cultured Hepa WT cells one day before drug treatment: seven mouse *CYP1A1* promoter-reporter constructs, each of which contains one DRE site, the control vector pGL3-promoter (**A**) or (**B**) pCYP1A1W-Luc, which contains the 1.4-kb *CYP1A1* promoter sequences, pCYP1A1-M1-Luc and pCYP1A1-M2-Luc, which contain mutations of the DRE at position −981 (DRE4), and the control vector pGL3-basic. After one day of treatment with TCDD (10^−9^ M) or with solvent alone at 0.1% (control), luciferase assays were performed to determine the promoter activity of mouse CYP1A1. ^*^ *p* < 0.05, the difference from solvent-treated cells (control) by one-way ANOVA with Tukey’s test.

Seven putative DREs are located in the ~1.4 kb promoter region of *CYP1A1*, whose transcription efficiency remains to be elucidated in response to TCDD. It is generally believed that as long as there is a DRE site at the promoter sequence, transcription can be initiated in response to TCDD. However, increasing evidence shows that mere DRE presence does not mean that the gene is inducible by TCDD, which suggests that although some DREs can be bound by the AhR complex, transcription may not be initiated. In order to elucidate this point, we cloned all seven DRE regions and their surrounding sequences (25 bp total) and inserted them into the reporter gene plasmid (pGL3-promoter) for investigation. We found that the transcriptional efficiency of these seven DREs differ greatly (Figure 3A). DRE4 has the highest efficiency of transcription, while DRE2 and DRE3 do not activate transcription. In order to verify the results of truncation, we performed *in situ* mutation of the DRE4 and got two vector mutants (pCYP1A1-M1-Luc, pCYP1A1-M2-Luc). We could see that mutation of DRE4 did decrease the transcription (Figure 3B). However, there are still great inductions compared with that of the control (pGL3), so the synergistic effects of DREs do play vital roles in transcriptions induced by TCDD.

### 2.4. The DRE Direction and Its Adjacent Sequences Can Affect the Efficiency of Transcription in Response to TCDD

**Figure 4.**
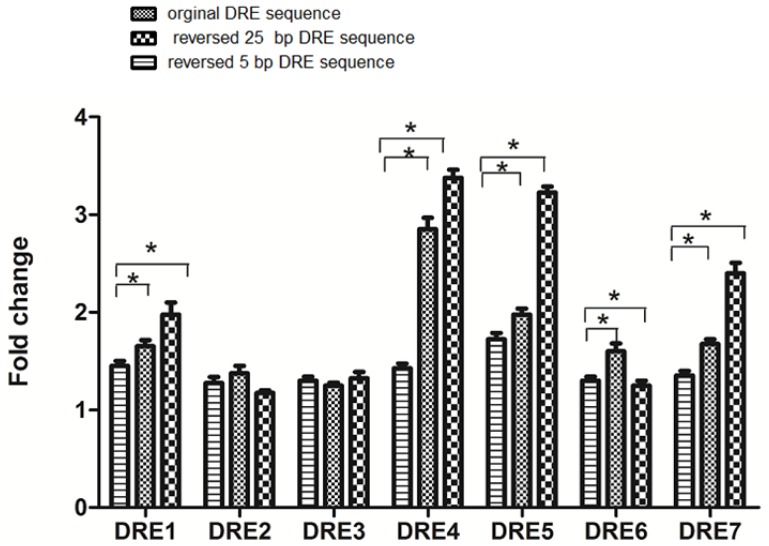
The DRE orientation and its adjacent sequences can affect the efficiency of transcription in response to TCDD. The mouse *CYP1A1* promoter-reporter constructs consisting of a 25-bp region surrounding the core DREs in the original orientation, reverse orientation or the reversed core sequence only with surrounding sequences in the original orientation, were transiently transfected into cultured Hepa WT cells. The pGL3-promoter vector is used as the control. One day later, the transfected cells were incubated with TCDD (10^−9^ M) or with solvent alone at 0.1% (control) for one day, followed by luciferase assays to determine the promoter activity. ^*^ *p* < 0.05, the difference from solvent-treated cells (control) by one-way ANOVA with Tukey’s test.

There has been a report that one DRE at the 5′ flanking region of mouse *CYP1A1* can activate a heterologous promoter and will function in either orientation [26]. Although the interaction of the AhR complex with the DRE occurs with the conserved “core” sequence, the DRE adjacent sequences also contribute to the specificity of DRE binding [31] and subsequent gene expression, since a single cloned DRE alone does not confer reporter activity [20,21]. Therefore, we systematically studied the orientations and adjacent sequences of the DREs. We found that when the substitution intolerant core sequences of each of the seven DREs were inverted, the transcriptional efficiency was generally reduced (Figure 4). However, when the 25-bp regions containing each DRE sequence were reversed, the transcriptional efficiencies of DRE1, DRE4, DRE5, DRE7 were enhanced, while the transcription of DRE6 was reduced. As mentioned in the preceding section, DRE2 and DRE3 did not activate transcription. We also did not see any significant changes of transcription activated by their reversed DRE sequences. The core sequence of DRE1, DRE4, DRE5 and DRE7 is 5′-CACGCNA-3′ and the core sequence of DRE6 is 5′-TNGCGTG-3′. Based on our experiments here, we suggest that the core DRE sequence with the orientation, 5′-TNGCGTG-3′, activates the transcription more efficiently than the core DRE in the opposite orientation (5′-CACGCNA-3′) when triggered by TCDD.

### 2.5. The Nucleotide “N” in the Core DRE (5′-TNGCGTG-3′) Sequence Influenced the Transcription Triggered by TCDD

**Figure 5.**
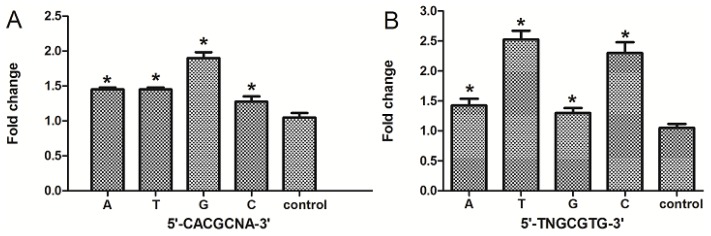
The nucleotide “N” in the core DRE (5′-TNGCGTG-3′) sequence contributed to the AhR-dependent transcriptional differences. The mouse *CYP1A1* promoter-reporter constructs, which contain “N” mutations of the DRE sequence at position −488 (5′-CACGCGA-3′) (**A**) or which contain “N” mutations of the reversed 25-bp DRE sequence at position, −488 (5′-TCGCGTG-3′) (**B**) were transiently transfected into cultured Hepa WT cells one day before drug treatment. The pGL3-basic vector is used as the control. After one day of treatment, luciferase assays were performed to determine the promoter activity of mouse CYP1A1. The transfected cells were incubated with TCDD (10^−9^ M) or with solvent alone at 0.1% (control). ^*^ *p* < 0.05, the difference from solvent-treated cells (control) by one-way ANOVA with Tukey’s test.

The consensus DRE sequence is 5′-TNGCGTG-3′, in which the “N” can be any one of the four nucleotides (A, C, T, G). To determine whether the different choices of nucleotide “N” in the core (5′-TNGCGTG-3′ or 5′-CACGCNA-3′) affects the efficiency of transcription, we compared the transcriptional difference corresponding to different combinations using the DRE at position −488. In cells transfected with the vector containing the 25-bp DRE original sequences (5′-CACGCGA-3′), our results showed that when the nucleotide “N” (the sixth nucleotide in the core sequence) is guanine, the transcription regulated by AhR was the highest compared with those containing one of the other three nucleotides. There was no significant difference in transcription when the nucleotide was adenine or thymine (Figure 5A). In cells transfected with vector containing reversed 25-bp DRE sequences (5′-TCGCGTG-3′), we found that when the nucleotide “N” was cytosine or thymine, the transcription efficiency was higher than if guanine or adenine were present. In other words, DRE sequences 5′-TTGCGTG-3′ or 5′-TCGCGTG-3′ have better transcriptional efficiency in response to TCDD than 5′-TGGCGTG-3′ or 5′-TAGCGTG-3′.

## 3. Discussion

Our results showed that DRE orientation, DRE adjacent sequences and the nucleotide “N” in the core DRE (5′-TNGCGTG-3′) sequence all have effects on transcription in response to TCDD. The genomic structure and position of the DREs cluster region in the mouse *CYP1A1* promoter are conserved in cattle, dog and rat genomes. These results suggest that the DREs cluster is important in mammalian biology [19]. Our results also showed that the location, core sequences and adjacent sequences of the seven DREs in the ~1.4 kb promoter were conserved in mouse and rat, and five of these are also present in the human CYP1A1 promoter.

AhR-Arnt complex binding, including increased chromatin accessibility at the enhancer, are not sufficient to induce *CYP1A1* expression. Induction also requires communication between the enhancer and promoter, which is mediated by the *C*-terminal region of AhR [32]. In order to study the transcription of seven DREs independently, the adjacent sequences must be considered; so, a 25-bp sequence containing the core DRE was selected for investigation. We found that DRE1, DRE4, DRE5, DRE6 and DRE7 could activate the transcription independently and DRE4 had the highest efficiency. Moreover, the results were confirmed by mutation. Our results were consistent with the previous report that DRE4 is fully conserved in position and sequence among mouse, rat and human promoter sequences, and all of them show AhR binding activity *in vitro* [29]. There has been reports that a single mouse *CYP1A1* DRE in either orientation can activate transcription and that the DRE adjacent sequences contribute to the specificity of DRE binding [29,31]. Our results showed that the core DRE sequence (5′-TNGCGTG-3′) activates transcription more efficiently than that of the core DRE (5′-CACGCNA-3′) in response to TCDD. Furthermore, the sequences around the DRE contribute greatly in activating transcription. The classical recognition motif of the AhR/ARNT complex contains the substitution intolerant core sequence, 5′-GCGTG-3′, within the consensus sequence, 5′-T/GNGC GTGA/CG/CA-3′, in the promoter region of AhR responsive genes [33]. The nucleotide “N” in the core DRE sequence (5′-TNGCGTG-3′) was also shown to be important for DRE-dependent transcription. When the nucleotide “N” is thymine or cytidine (T, C), the transcription efficiency was relatively stronger compared with that of the adenine or guanine (A, G). In all, we found that the seven DREs have different transcriptional efficiency in response to TCDD, and the difference may be closely related to their orientations, adjacent sequences and the nucleotide “N” in the core DRE sequence. Our study lays a good foundation for further investigation into the transcriptional regulation triggered by dioxin and dioxin-like compounds.

## 4. Materials and Methods

### 4.1. Materials

Luciferase reporter vectors, pGL3-Basic vector and pGL3-promoter vector were purchased from Promega (Fitchburg, WI, USA). High fidelity polymerase was purchased from the Kang Company (Beijing, China). Dimethyl sulfoxide (DMSO) was purchased from Sigma (St. Louis, MO, USA). 2,3,7,8-TCDD was purchased from Wellington Laboratories Inc. (Ontario, ON, Canada). A dual luciferase reporter gene assay kit was purchased from Promega (Fitchburg, WI, USA). Alpha modified minimum essential medium (α-MEM) and fetal bovine serum (FBS) were purchased from Invitrogen (Carlsbad, CA, USA).

### 4.2. Cell Culture and Chemical Treatment

The Hepa WT, a cell line derived from mouse liver cancer cells, was purchased from the American Type Culture Collection (ATCC). Cells were maintained in medium with 10% FBS and incubated at 37 °C in a water-saturated 5% CO_2_ incubator. All reagents for the cell culture were from Invitrogen (Carlsbad, CA, USA). The most potent congener of dioxins, TCDD, was employed at low concentrations of 10^−9^ M. The solvent, dimethyl sulfoxide (DMSO), was present at 0.1% for all treatments. Treated cultures were compared with cultures exposed to 0.1% DMSO alone.

### 4.3. Plasmid Construction

pCYP1A1W-Luc consists of the mouse *CYP1A1* promoter sequences (0–1400 bp) upstream of a firefly luciferase gene in pGL3-basic vectors (Promega, Madison, WI, USA). pCYP1A1W-Luc was constructed using sense primer 5′-CAC GCT CGA GAA CAG GTT GAG TTA GAC-3′ and antisense primer 5′-CAC GAA GCT TCA GGG TTA GGG TGA AG-3′. *Xho*I and *Hind*III restriction sites were added at the 5′ ends of sense and antisense primers, respectively. The deletion constructs, pCYP1A1-T1-Luc, which contains only one DRE at site −488, was constructed using sense primer 5′-TAT AGA GCT CAG CGC GAA CTT CGG CCG ATA-3′ and antisense primer 5′-CAC GAA GCT TCA GGG TTA GGG TGA AG-3′. pCYP1A1-T2-Luc, which contains no DRE, was constructed using sense primer 5′-CCT CGA GCT CGT AGG CAA GAG GAT CTT AC-3′ and antisense primer 5′-CAC GAA GCT TCA GGG TTA GGG TGA AG-3′. *Sac*I and *Hind*III restriction sites were added at the 5′ ends of the sense and antisense primers, respectively. The mutated construct, pCYP1A1-M-Luc, derived from pCYP1A1W-Luc, was constructed by site-directed mutagenesis (position −981), accomplished using mutagenic primers. The mutagenic primers include sense primer, 5′-CCT CCA GGC TCT GAA TTC AAC TCC GGG GCA CC-3′, and anti-sense primer, 5′-GGT GCC CCG GAG TTG AAT TCA GAA GAG CCT GGA GG-3′, where the original sequence of the putative DRE (5′-CACGC-3′) was replaced by an *EcoR*I restriction site (5′-GAATTC-3′). The other 21 vectors with different combinations of DRE mutations were constructed in pGL3-promoter vectors, using 3′ antisense primer 5′-CCA CAA GCT TTT TGC AAA AGC CTA GGC CTC C-3′ and the sense primers listed in the Supplementary Information.

### 4.4. Transient Transfection

Cultured cells were seeded in 24-well plates at 40,000 cells per well 24 h before being transfected transiently with purified plasmids (0.5 μg per well) and PolyJet™ reagent (SignaGen Laboratories, Rockville, MD, USA), according to the manufacturer’s instructions. Renilla luciferase plasmids were co-transfected for normalization. The transfection efficiency was about 15%.

### 4.5. Luciferase Assay

For luciferase measurement, sample wells were washed twice with phosphate-buffered saline, followed by the addition of cell lysis buffer (Promega) and shaking of the plates for 20 min at room temperature to allow cell lysis. Insoluble material was removed by centrifugation, and 50-μL aliquots of the resulting lysates were transferred to white 96-well microplates for the measurement of luciferase activity. Activities of the enzyme were measured with the Promega Dual-Luciferase Reporter Assay System (Promega, Fitchburg, WI, USA). Firefly luciferase activity was determined by the addition of 50 μL of Luciferase Assay Reagent II and measurement of the resulting luminescence using a TECAN infinite f200 pro luminometer (Tecan Group Ltd., Männedorf, Switzerland) with automatic injection of Promega stabilized luciferase reagent. Renilla luciferase activity was subsequently determined following the addition of 100 μL of Stop & Glow Reagent to the same reaction tube. Firefly luciferase activity was expressed relative to that of Renilla luciferase.

### 4.6. Statistics

Statistical tests were made by the GraphPad Prism (version 5, La Jolla, CA, USA). All statistical analyses were done using one-way ANOVA, followed by Tukey’s test, to determine the significance of differences between samples. Significance is presented as *p* < 0.05 compared with the appropriate controls.

## 5. Conclusions

We found that DRE orientation, DRE adjacent sequences and the nucleotide “N” in the core DRE (5′-TNGCGTG-3′) sequence could all contribute to the AhR-regulated *CYP1A1* transcription in response to TCDD. To our knowledge, this is the first study to report systematically the function of the dioxin response elements (DREs) of the murine *CYP1A1* gene promoter.

## Supplementary Information
