# Supplementary material for: Functional Analysis of the Dioxin Response Elements (DREs) of the Murine CYP1A1 Gene Promoter: Beyond the Core DRE Sequence
Source: Int J Mol Sci. 2014 Apr 16;15(4):6475–87. doi: 10.3390/ijms15046475 (PMC4013641; doi:10.3390/ijms15046475)
Supplement: Supplementary file 1 [file ijms-15-06475-s001.pdf]

Supplementary Information

Table S1. Primers for constructing vectors containing original DRE sequences.

DRE site	Sequence of the sense primer
-1379	5'-TTAAGGTACCTGAGTTAGACACGCCAAGTTCAGATGAGCTCGGCCAACAGA-3'
-1203	5'-TTAAGGTACCCGAGCATCGCACGCAAACCCGGCCCGAGCTCGGCCAACAGAC-3'
-1058	5'-TTAAGGTACCTCCCCCAGCTAGCGTGACAGCACTGGAGCTCGGCCAACAGAC-3'
-981	5'-TTAAGGTACCGGCTCTTCTCACGCAACTCCGGGGCGAGCTCGGCCAACAGAC-3'
-892	5'-TTAAGGTACCAGGCTTACGCACGCTAGCCTCAGGAGAGCTCGGCCAACAGAC-3'
-821	5'-TTAAGGTACCCCTGCTGTCTCGCGTGGATCCTTCCGAGCTCGGCCAACAGAC-3'
-488	5'-TTAAGGTACCTGCGCTTCTCACGCGAGCTTGGACTGAGCTCGGCCAACAGAC-3'

Table S2. Primers for constructing vectors containing inverted 25 bp original DRE sequences.

DRE site	Sequence of the sense primer
-1379	5'-TTAAGGTACCATCTGAACTTGGCGTGTCTAACTCAGAGCTCGGCCAACAGAC-3'
-1203	5'-TTAAGGTACCGGGCCGGGTTTGCGTGCGATGCTCGGAGCTCGGCCAACAGAC-3'
-1058	5'-TTAAGGTACCCAGTGCTGTCACGCTAGCTGGGGGAGAGCTCGGCCAACAGAC-3'
-981	5'-TTAAGGTACCGCCCCGGAGTTGCGTGAGAAGAGCCGAGCTCGGCCAACAGAC-3'
-892	5'-TTAAGGTACCTCCTGAGGCTAGCGTGCGTAAGCCTGAGCTCGGCCAACAGAC-3'
-821	5'-TTAAGGTACCGGAAGGATCCACGCGAGACAGCAGGGAGCTCGGCCAACAGAC-3'
-488	5'-TTAAGGTACCAGTCCAAGCTCGCGTGAGAAGCGCAGAGCTCGGCCAACAGAC-3'

Table S3. Primers for constructing vectors containing inverted core DRE sequences.

DRE site	Sequence of the sense primer
-1379	5'-TTAAGGTACCTGAGTTAGATGGCGTGAGTTCAGATGAGCTCGGCCAACAGAC-3'
-1203	5'-TTAAGGTACCCGAGCATCGTTGCGTGACCCGGCCCGAGCTCGGCCAACAGAC-3'
-1058	5'-TTAAGGTACCTCCCCCAGCCACGCTAACAGCACTGGAGCTCGGCCAACAGAC-3'
-981	5'-TTAAGGTACCGGCTCTTCTTTGCGTGCTCCGGGGCGAGCTCGGCCAACAGAC-3'
-892	5'-TTAAGGTACCAGGCTTACGTAGCGTGGCCTCAGGAGAGCTCGGCCAACAGAC-3'
-821	5'-TTAAGGTACCCCTGCTGTCCACGCGAGATCCTTCCGAGCTCGGCCAACAGAC-3'
-488	5'-TTAAGGTACCTGCGCTTCTTCGCGTGGCTTGGACTGAGCTCGGCCAACAGAC-3'

Table S4. Primers for constructing DRE mutants at position -488.

Base mutation	Sequence of the sense primer
“N” mutation of original DRE sequence at position -488	
G→C	5'-TTAAGGTACCTGCGCTTCTCACGCCAGCTTGGACTGAGCTCGGCCAACAGAC-3'
G→A	5'-TTAAGGTACCTGCGCTTCTCACGCAAGCTTGGACTGAGCTCGGCCAACAGAC-3'
G→T	5'-TTAAGGTACCTGCGCTTCTCACGCTAGCTTGGACTGAGCTCGGCCAACAGAC-3'
“N” mutation of inverted 25 bp original DRE sequences at position -488	
C→G	5'-TTAAGGTACCAGTCCAAGCTGGCGTGAGAAGCGCAGAGCTCGGCCAACAGAC-3'
C→A	5'-TTAAGGTACCAGTCCAAGCTAGCGTGAGAAGCGCAGAGCTCGGCCAACAGAC-3'
C→T	5'-TTAAGGTACCAGTCCAAGCTTGCGTGAGAAGCGCAGAGCTCGGCCAACAGAC-3'
“N” mutation of inverted core DRE sequences at position -488	
C→G	5'-TTAAGGTACCTGCGCTTCTTGGCGTGGCTTGGACTGAGCTCGGCCAACAGAC-3'
C→A	5'-TTAAGGTACCTGCGCTTCTTAGCGTGGCTTGGACTGAGCTCGGCCAACAGAC-3'
C→T	5'-TTAAGGTACCTGCGCTTCTTTGCGTGGCTTGGACTGAGCTCGGCCAACAGAC-3'
